# Reduced cellularity of bone marrow in multiple sclerosis with decreased MSC expansion potential and premature ageing in vitro

**DOI:** 10.1177/1352458517711276

**Published:** 2017-05-26

**Authors:** Juliana Redondo, Pamela Sarkar, Kevin Kemp, Paul F Virgo, Joya Pawade, Aimie Norton, David C Emery, Martin G Guttridge, David I Marks, Alastair Wilkins, Neil J Scolding, Claire M Rice

**Affiliations:** School of Clinical Sciences, University of Bristol, Bristol, UK; School of Clinical Sciences, University of Bristol, Bristol, UK; School of Clinical Sciences, University of Bristol, Bristol, UK; Department of Immunology, Southmead Hospital, Bristol, UK; Department of Pathology, Southmead Hospital, Bristol, UK; Department of Pathology, Southmead Hospital, Bristol, UK; School of Clinical Sciences, University of Bristol, Bristol, UK; NHS Blood and Transplant, Bristol, UK; Blood and Marrow Transplant Unit, University Hospitals Bristol NHS Foundation Trust, Bristol, UK; School of Clinical Sciences, University of Bristol, Bristol, UK; School of Clinical Sciences, University of Bristol, Bristol, UK; School of Clinical Sciences, University of Bristol, Bristol, UK

**Keywords:** Cell therapy, bone marrow, mesenchymal stromal cell, multiple sclerosis

## Abstract

**Background:**

Autologous bone-marrow-derived cells are currently employed in clinical studies of cell-based therapy in multiple sclerosis (MS) although the bone marrow microenvironment and marrow-derived cells isolated from patients with MS have not been extensively characterised.

**Objectives:**

To examine the bone marrow microenvironment and assess the proliferative potential of multipotent mesenchymal stromal cells (MSCs) in progressive MS.

**Methods:**

Comparative phenotypic analysis of bone marrow and marrow-derived MSCs isolated from patients with progressive MS and control subjects was undertaken.

**Results:**

In MS marrow, there was an interstitial infiltrate of inflammatory cells with lymphoid (predominantly T-cell) nodules although total cellularity was reduced. Controlling for age, MSCs isolated from patients with MS had reduced in vitro expansion potential as determined by population doubling time, colony-forming unit assay, and expression of β-galactosidase. MS MSCs expressed reduced levels of Stro-1 and displayed accelerated shortening of telomere terminal restriction fragments (TRF) in vitro.

**Conclusion:**

Our results are consistent with reduced proliferative capacity and ex vivo premature ageing of bone-marrow-derived cells, particularly MSCs, in MS. They have significant implication for MSC-based therapies for MS and suggest that accelerated cellular ageing and senescence may contribute to the pathophysiology of progressive MS.

## Introduction

Bone-marrow-derived stem cells, including mesenchymal stromal cells (MSCs), modulate a range of processes relevant to inflammatory demyelinating disease including immunoregulation, inflammation, neurotrophin production, apoptosis, angiogenesis, endogenous neurogenesis and oligodendrogenesis, as well as oligodendrocyte migration and remyelination. These properties, combined with their favourable safety profile, have facilitated early clinical translation of MSC-based cell therapies for a range of conditions including chronic neurological diseases such as multiple sclerosis (MS).^1^

Although clinical studies of MSC-based therapy are already underway in MS, relatively little information is available regarding the bone marrow microenvironment,^2^ or phenotype of MSCs and other marrow-derived stem cell populations in MS.^3–7^ Indeed, it is not clear whether previous immunotherapy or the inflammatory environment in MS could compromise stem cell function.^8^ The few studies available have generally been small and comparators have included those with underlying haematological malignancies,^2^ cardiac disease^9^ and bone disease.^4,5^ They have not always been adequately controlled for age and time in vitro.

The aim of this study was to characterise the bone marrow microenvironment in MS and determine whether the expansion potential of MS MSCs is comparable to that of control MSCs. Crucially, our data analysis employed a multiple regression model to allow for independent effects of age, passage number, and presence or absence of disease on proliferation and senescence.

## Materials and methods

### Bone marrow collection

Control bone marrow samples for isolation of MSCs were obtained at the time of elective total hip replacement for the indication of osteoarthritis courtesy of the Orthopaedic Department, Southmead Hospital (UK Research Ethics Committee (REC) 10/H102/69). Individuals with a history of immune disease or immunotherapy were excluded. Bone marrow samples from MS patients were obtained from participants in the ‘SIAMMS-II’ (NCT01932593; UK REC 13/SW/0255)^10^ and ‘ACTiMuS’ trials (NCT01815632; UK REC 12/SW/0358).^11^ Both trials include only participants with progressive MS, and the ‘ACTiMuS’ trial has an additional requirement for progression to have occurred within the 12 months preceding trial entry. The MS cohort were younger (*n* = 28, median age = 51 years, mean = 51.9 years; control cohort: *n* = 11, median age = 59 years, mean = 59.7 years; Student’s *t*-test: *p* = 0.003). For full cohort details, see Supplementary Information (Table 1). Age was not associated with duration of disease progression (Spearman’s *r* = 0.277, *p* = 0.153). No participants with primary progressive MS (*n* = 10) had been exposed to disease-modifying therapy (DMT). Of the 18 participants with secondary progressive MS, 8 had been exposed to beta-interferon and/or glatiramer and one had also been treated with alemtuzumab. For the experiments examining MSC phenotype in vitro, only two participants with secondary progressive disease had received beta-interferon, or beta-interferon and glatiramer and none had been exposed to alemtuzumab or other DMT.

**Table 1.** Cohort characteristics, cellularity, immunophenotype, proliferation and senescence of bone marrow in MS.

	MS	Age (years)	Marrow trephine						Marrow aspirate		
			Cellularity (%)	CD20 (%, *n* nodules)	CD3 (%, *n* nodules)	Ki67 (%)	p16 cellular areas	p16 hypocellular areas	CD34 × 10^6^/kg	TNC × 10^8^/kg	MNC × 10^8^/kg
1	PP	48	40	<5, 0	<5, 0	90	n/a	n/a	0.55	1.14	0.22
2	SP	33	60	<5, 0	<10, 3	50	++	+	1.1	1.64	0.25
3	PP	47	40	<10, 0	10, 0	25	+++	++	1.05	1.22	0.2
4	PP	47	40	>10, 0	15, 8	90	++	+	0.3	1	0.22
5	PP	52	40	5–10, 0	<5, 0	30	++	++	0.74	0.68	0.21
6	SP	59	30	<5, 1	<5, 0	60	++	+	0.49	0.81	0.17
7	SP	55	40	5, 0	<10, 4	30	++	++	0.45	0.89	0.14
8	SP	39	40	10, 0	15, 0	20	++	+	0.71	0.77	0.18
9	SP	56	30	5, 0	10, 0	30	+	+	0.75	0.85	0.15
10	SP	53	50	10, 0	15, 3	70	+++	++	0.61	1.04	0.27
11	PP	49	45	10, 0	10, 2	20	++	+	0.63	0.62	0.16
12	PP	64	40	5, 0	10, 3	40	++	+	0.31	0.66	0.13
13	PP	50	40	<10, 0	5, 0	70	++	+	0.38	0.5	0.08
14	SP	50	50	<5, 0	15, 8	80	+++	++	0.98	1.36	0.46
15	SP	63	30	<1, 0	<5, 0	60	+	+	0.59	1.06	0.17
16	SP	55	50	<10, 0	20, 0	80	++	+	0.66	0.66	0.24
17	SP	41	50	10, 0	20, 0	60	++	++	0.82	1.66	0.26
18	SP	50	40	<10, 0	15, 1	80	++	+	0.61	0.77	0.16
19	SP	49	50	>20, 5	>20, 6	70	+++	++	1.2	1.02	0.23
20	SP	58	30	<5, 0	>15, 5	60	++	+	0.49	0.63	0.15
21	SP	57	40	<5, 0	>15, 12	80	++	++	0.8	1.42	0.25
22	SP	62	25	<5, 1	<10, 2	60	+++	+	0.18	1.25	0.3
23	SP	55	45	<10, 0	<5, 2	40	+++	++	1.95	1.77	0.29
Mean (SD)			41.09 (8.39)			56.30 (22.77)			0.71 (0.37)	1.02 (0.36)	0.21 (0.08)
Median (range)			40 (25–60)			60 (25–60)			0.63 (0.18–1.95)	1 (0.5–1.77)	0.21 (0.08–0.46)

PP: primary progressive multiple sclerosis; SP: secondary progressive multiple sclerosis; TNC: total nuclear cell count; MNC: mononuclear cell count; SD: standard deviation.

+ indicates 1 to 4 positive cells; ++ indicates 5 to 10 positive cells; +++ indicates 11 to 15 positive cells per high power field (×40 magnification).

### Bone marrow trephine immunohistochemistry

Trephine sections were cut (haematoxylin and eosin = 1 µm, immunohistochemistry = 2 µm and reticulocytes = 3 µm), mounted and stained (Supplementary Information Table 2) following formalin fixation, decalcification and paraffin-embedding. Reporting criteria are presented in the Supplementary Information.

### Bone marrow harvest analysis

All bone marrow collections were evaluated for cell viability, total mononuclear cell count (MNC) and specific viable CD34 count.^12^

### Isolation of bone-marrow-derived MSCs

Control bone marrow from the femoral shaft was collected in RPMI medium (Sigma) with 1000-IU heparin. Samples from patients with MS were aspirated from the posterior iliac crest during bone marrow harvest and collected in heparin before being transported to the laboratory in EDTA (K2) tubes. Subsequently, marrow samples were processed in an identical manner and MSCs were isolated from both control and MS-affected marrow using density gradient centrifugation as previously described.^5,13^

### MSC differentiation and immunophenotype

To ensure isolated MSCs conformed to international defining criteria,^14^ cell surface immunophenotype, as well as adipogenic, osteogenic and chondrogenic differentiation potential of MSCs, was examined.^5^

### Immunocytochemistry

Immunocytochemistry was performed as previously described,^13^ and details of antibodies used are presented in the Supplementary Information (Table 2).

### Population doubling time

Population doubling time (PDT) = (*CT* × ln2)/(*N*/*N*_0_), where *CT* is the time in culture, *N* is the final number of cells and *N*_0_ is the initial number of cells seeded.

### Colony-forming unit assay

Colony-forming unit (CFU) assay was performed at p1, p3 and p5. Cells were treated with trypsin and seeded in 6-well plates at 250, 125, 62, 31 and 15 cells/well. After 14 days, fixed cells were stained with 0.5% crystal violet in methanol (Sigma). Calculation of CFU efficiency was performed dividing number of colonies by the total number of cells seeded, ×100.

### Senescence-associated β-galactosidase staining

At p7, p10 and p12, MSCs were seeded at 5 × 10^4^ cells/35-mm wells and stained at 24 hours for senescence-associated β-galactosidase (SA-β-gal staining kit, Cell Signaling Technology). The percentage of SA-β-gal-positive cells in five random fields was calculated.

### DNA extraction

Genomic DNA was extracted from 2 × 10^6^ control and MS MSCs at p2 and p6 using the GenElute Mammalian Genomic DNA Miniprep Kit (Sigma).

DNA concentrations were determined using a Qubit® Fluorometer and Quant-iT^™^ DNA assay kit (Invitrogen) to ensure equal sample loading.

### Telomere length assay

TeloTAGGG telomere length assay (Roche) was used according to manufacturer’s instructions. Average size distribution of terminal restriction fragments (TRF) was calculated using the following formula: TRF = Σ(*OD*)/ Σ(*OD*/*L*), where *OD* is the chemiluminescent signal and *L* is the length of TRF obtained comparing the location of TRF on the blot relative to a molecular weight standard.

### Statistical analysis

Unless otherwise stated, statistical analysis employed a multiple regression model (STATA v12; StataCorp) which, where appropriate, allowed for correlation between replicates performed using cells isolated from the same individual (cluster option). Non-parametric bootstrap analysis was used to estimate standard errors (SEs) and confidence intervals (CI) to account for possible non-normality of the parameter’s distribution. All graphs were generated using GraphPad PRISM 5^™^ (GraphPad Software) which was also used for statistical analyses other than multiple regression analyses. Unless otherwise stated, bar graphs show mean ± SE of the mean, and regression lines are fitted with 95% CIs. For all analyses, values of *p* < 0.05 were considered statistically significant.

## Results

### Morphology, cellularity and fibrosis of MS bone marrow microenvironment

All analysed trephines (*n* = 23) were adequate in size and integrity with at least six interstitial spaces; small or disrupted specimens were not considered.

Bone marrow trephines from patients with MS contained the expected range of myeloid and erythroid precursors, and colonies were well formed with normal maturation and megakaryocytic morphology and distribution.^15^ The myeloid:erythroid ratio (M:E) was normal (3–4:1) with only a single patient having a decreased ratio (2:1, 4.3%). Bone marrow fibrosis was not detected, and the bone structure was generally within the expected limits although four (17.4%) patients were noted to have more prominent bone remodelling/trabeculae.

**Figure 1.**
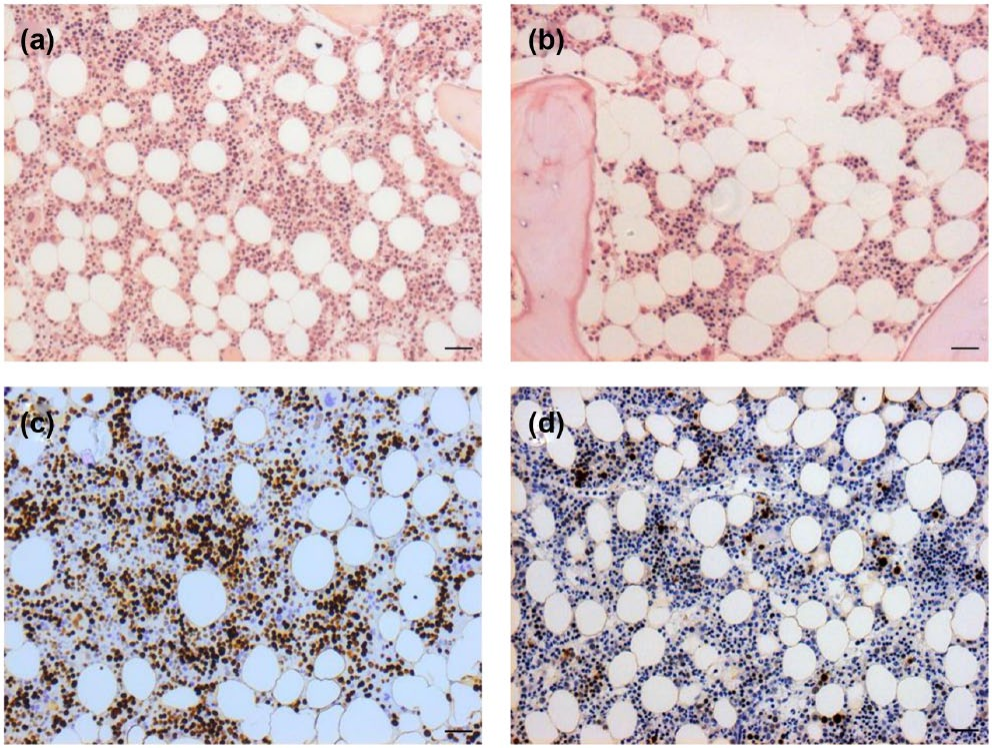
Cellularity and cell proliferation in marrow from subjects with MS. (a, b) MS trephine with cellularity and (c, d) cell proliferation as determined by Ki67 expression (a, c) considered normal for age (50 years) and (b) hypocellular (40% instead of 60%) with (d) reduced proliferation (20%) from a participant aged 39 years. Scale bar: 100 µm.

Bone marrow cellularity was lower than expected for age in almost half the patients with progressive MS (*n* = 11, 47.8%), and of these five patients, 22% had severe marrow hypoplasia (⩽30% cellularity) not in keeping with age (Table 1, Figure 1(b)).

Within the MS cohort, Ki67 expression varied over a wide range (20%–90%, Table 1; Figure 1(c) and (d)) although the majority of samples showed decreased proliferation as assessed by values ⩽60% (*n* = 14, 60.9%). As expected, erythroid cells demonstrated higher levels of proliferation (75%–90%) than myeloid cells (15%–80%). However, 11 MS specimens (47.8%) showed markedly reduced proliferation within the erythroid compartment (<70%) in the context of globally low levels of proliferation. Although the MS specimens had few proliferating megakaryocytes, the proportion was within the expected range (10%–25%).

### Immunological profile of MS bone marrow

Trephines from MS patients had normal expression of CD34+ haematopoietic cells, CD61+ megakaryocytes and CD138+ plasma cells. All specimens were negative for P53+ cells and natural killer cells (CD56).

**Figure 2.**
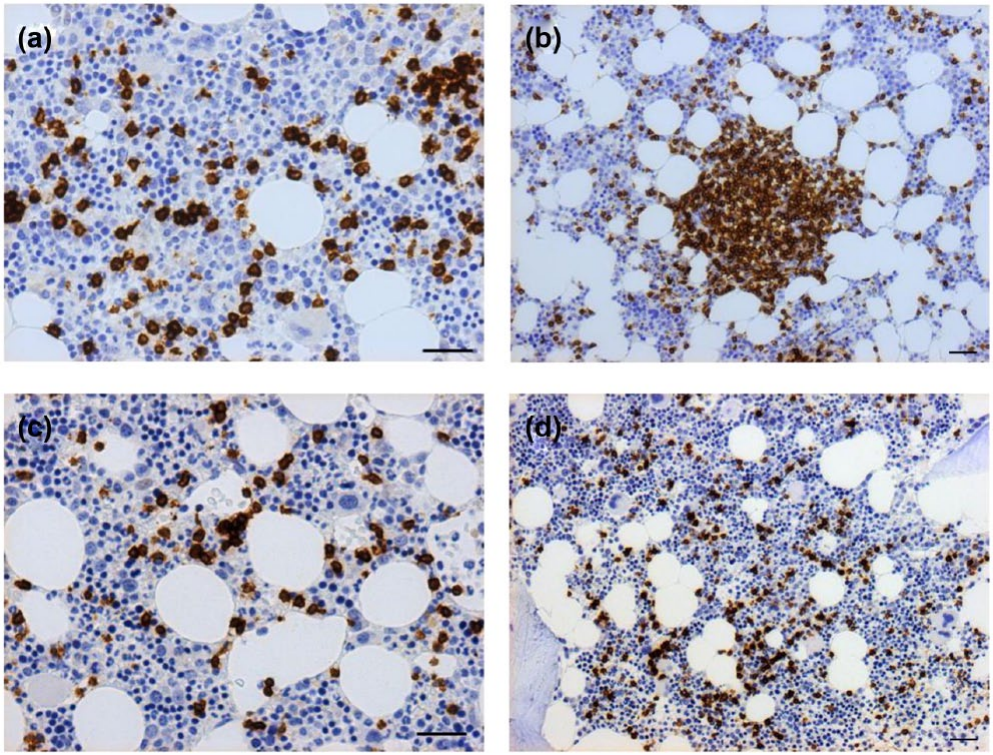
B- and T-cell infiltrates and nodules in bone marrow trephines from patients with MS. (a, b) Both CD3-positive and (c, d) CD20-positive infiltrates were noted. The majority of MS trephines had (b) T-cell nodules but few samples had CD20-positive nodules. Scale bar: 100 µm.

Infiltrates of T- and B-lymphocytes were assessed as percentage of overall cellularity using CD3 and CD20 expression (Figure 2). Infiltrates of T-lymphocytes accounted for 5%–20% of cellularity and B-lymphocytes accounted for 5%–10% (Table 1). The distribution of cells occurred as a dispersed interstitial infiltrate and lymphoid nodules (Figure 2).

The majority of MS specimens contained T-cell nodules (*n* = 13, 56.2%), whereas only three MS samples (13%) had B-cell nodules (Figure 2). Two had abnormal distribution of B-lymphoid tissue: one due to paratrabecular distribution of B-lymphoid nodule and one due to moderate interstitial infiltration. Neither patient had B-symptoms, abnormalities on full blood count or detectable paraprotein.

**Figure 3.**
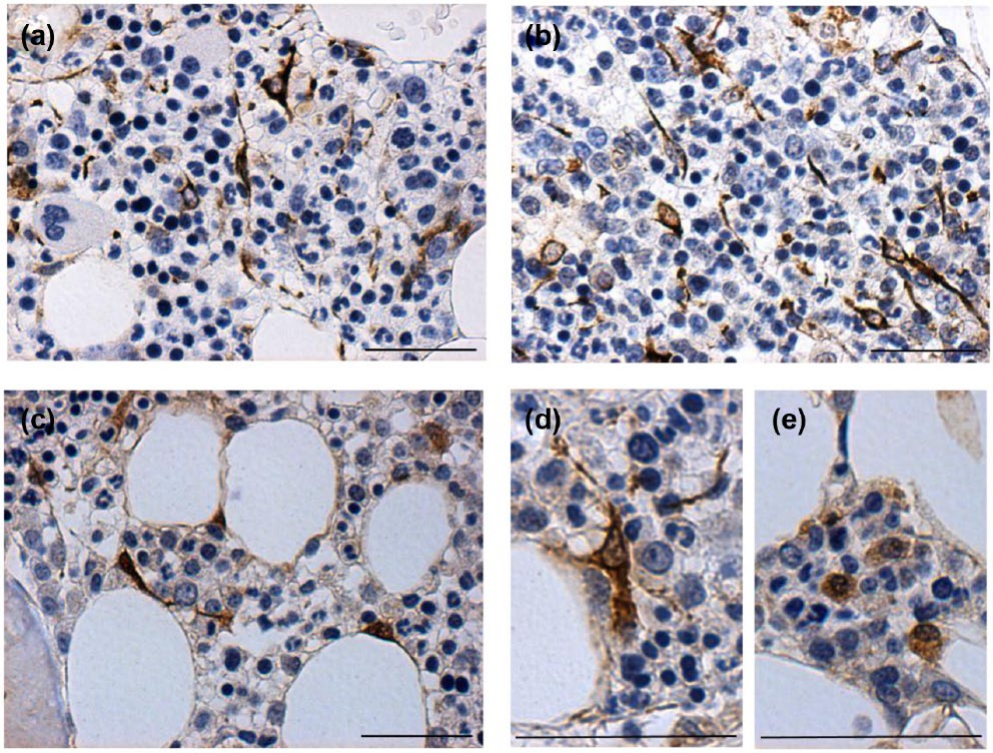
Vimentin and p16 expression in cells with stromal morphology. (a, b) Vimentin-positive cells with elongated, spindle-shaped morphology were noted to form a network within the marrow. (c, d) Cells expressing p16 in a predominantly nuclear distribution also had spindle-shaped morphology and were distributed within hypocellular areas, (e) while those with a more cytoplasmic pattern of staining were located in more cellular areas of marrow.

The connective tissue compartment incorporating blood vessels and stromal cells was analysed. The intermediate filament vimentin was present in elongated, spindle-shaped cells distributed as a network within the haematopoietic compartment (Figure 3(a) and (b)).

All MS samples demonstrated p16+ staining in cells with stromal morphology either as nuclear/perinuclear pattern or in a cytoplasmic distribution, and this varied with cellularity (Figure 3(c)–(e)). Cells expressing nuclear p16 tended to be spindle-shaped with a dense staining pattern and located in hypocellular areas, frequently adjacent to adipocytes/fat (Figure 3(c) and (d)). Those with cytoplasmic p16 were more rounded with weaker staining and were located in more cellular areas (Figure 3(e)).

### Bone marrow aspirate

Cell viability was >96% in all cases. Median total nuclear count (TNC) was 1 × 10^8^/kg and median CD34 cell count was 0.63 × 10^6^/kg (Table 1). As previously reported, CD34 count adjusted for weight declined with increasing age (*p* = 0.007).^16^ CD34 counts in primary progressive disease (mean = 0.57 × 10^6^/kg, median = 0.55 × 10^6^/kg) were lower than in secondary progressive disease (mean = 0.77 × 10^6^/kg, median = 0.69 × 10^6^/kg) but this was not significant when adjusted for age (*p* = 0.085). There was no significant association between CD34 count and duration of progression of MS.

### MSC isolation and immunophenotype

Bone-marrow-derived MSCs from both control (*n* = 11) and MS (*n* = 16) individuals displayed characteristics distinctive of MSCs in culture;^14^ cells were plastic-adherent with elongated, fibroblast-like morphology. Culture expanded MSCs from MS patients could be induced to undergo adipogenic, osteogenic and chondrogenic differentiation as previously described.^5^

Both control and MS MSCs displayed high expression of cell surface mesenchymal markers including CD90, CD105, CD73, CD271, CD44 and low expression of CD45 (data not shown). Immunocytochemistry confirmed expression of CD105, CD73, CD271, fibronectin and beta-III (BIII) tubulin in control and MS MSCs (data not shown).

### Expansion potential of MSCs declines with age and expansion in vitro

**Figure 4.**
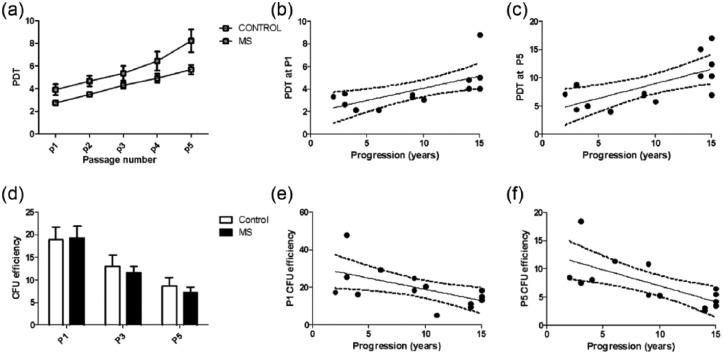
Increased PDT and decreased CFU of MS MSCs. (a) PDT increases with time in vitro for MSCs isolated from control subjects and patients with MS, (b, c) but accounting for this and the age of subjects, there is an independent effect of the presence of MS to increase PDT and this is positively associated with duration of progressive MS. Data from two MS cultures which failed to expand prior to p5 are not included in Figure 4(a) although the data were included in the multiple regression model. (d) MSC CFU number declines with time in vitro and, when age is taken into consideration, CFU number is decreased at all passages when MSCs are isolated from patients with progressive MS. When the confounding effect of age is taken into consideration, this was statistically significant. (e, f) There was also a negative association between CFU number and duration of progressive MS.

As expected, MSC expansion at higher passage (p) numbers was slow and associated with morphological changes previously reported to be associated with senescence including increased cell size and cytoplasmic granularity.^17^ To investigate the proliferation capacity of MSCs prior to expected senescence in vitro, PDT was calculated sequentially from p1 to p7 for MSCs isolated from 8 control subjects and 15 patients with MS. PDT increased with in vitro passage for both MS and control MSCs (Figure 4(a)) and PDT at p5 was significantly longer than PDT at p1 (comparison of PDT at p1 and p5 using Wilcoxon matched-pairs *t*-test, *p* < 0.0001).

Data were analysed using a multiple regression model with cluster analysis to allow for correlation between samples isolated from the same individual and effects of age, passage number and, where relevant, duration of progression of MS. Independent effects of age (*p* = 0.002), passage number (*p* < 0.0001) and presence of disease (*p* < 0.0001) were observed. In the MS cohort, collinearity was not observed between age and duration of MS disease progression and the latter had an independent, statistically significant effect on PDT (*p* = 0.012) (Figure 4(b) and (c)).

CFU assays were performed at p1, p3 and p5 using MSC cultures from 8 control subjects and 15 patients with MS. A decline in CFU was seen sequentially with increasing passage number (Figure 4(d)); comparison of CFU at p1 and p3, p3 and p5, and p1 and p5 was performed using Wilcoxon matched-pairs *t*- test and the difference was highly significant for each analysis (*p* < 0.0001).

Using the multiple regression model with cluster analysis, negative independent statistically significant effects of age (*p* < 0.001), presence of MS (*p* = 0.004) and passage number (*p* < 0.0001) were seen on CFU number. Within the MS cohort, there was an independent effect of duration of progression (*p* = 0.008) and collinearity with age was not observed (Figure 4(e) and (f)). There was no difference in CFU number in the samples isolated from those with primary or secondary progressive disease.

### MSC Stro-1 expression decreases with time in culture and duration of progressive MS

**Figure 5.**
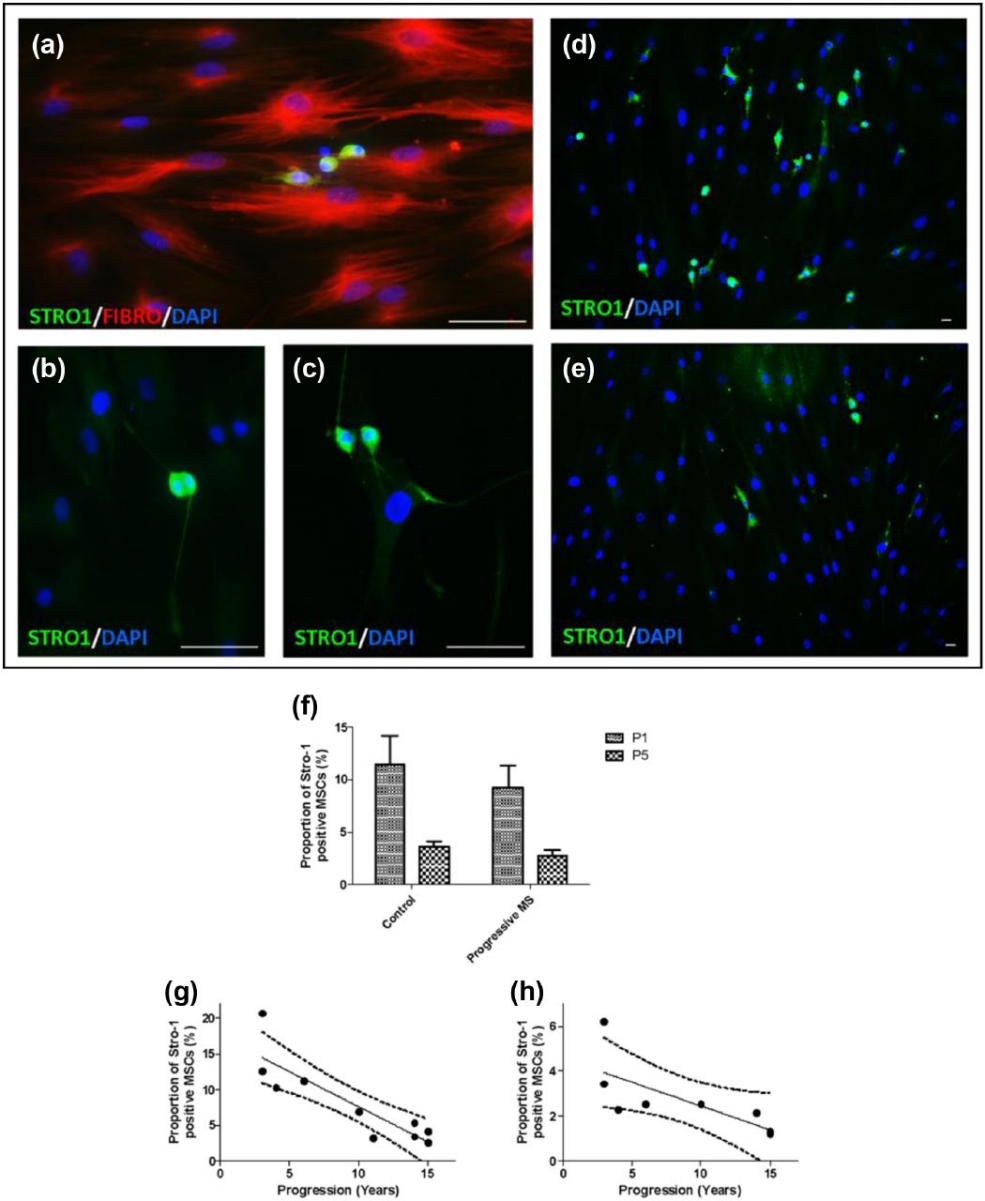
MSC expression of Stro-1 declines with expansion in vitro and in the presence of MS. MSCs are known to express fibronectin (Fibro) in vitro and a proportion of MSCs also express Stro-1. (a) Stro-1 expression was noted in small, round cells, (b, c) some of which were mitotically active. (d) The proportion of MSCs expressing Stro-1 was greater in MSCs isolated from control subjects (e) than in MS MSCs. Scale bar: 200 µm. (f) Expression of Stro-1 by MSCs decreases with time in vitro and, when age is accounted for, a smaller proportion of MS MSCs expressed Stro-1 compared to the proportion of control MSCs which were Stro-1 positive. There was a negative association between the proportion of MS MSCs expressing Stro-1 and duration of progressive MS at (g) p1 and (h) p5.

The expression of the early mesenchymal precursor marker Stro-1 was analysed in control and MS MSCs by immunocytochemistry at p1 and p5 (Figure 5; control: *n* = 6; MS: *n* = 10). Stro-1 expression was observed uniformly in a sub-population of small, round cells with small nuclei, some of which were noted to be mitotically active (Figure 5(a)–(c)). As expected, the proportion of Stro-1-positive cells declined between p1 and p5 (Figure 5(f); Wilcoxon matched pairs *t*-test, *p* = 0.0001).^18^ Multiple regression modelling with cluster analysis demonstrated independent negative effects of age (*p* < 0.0001), presence of MS (*p* = 0.001) and passage number (*p* < 0.0001) on Stro-1 expression. The proportion of Stro-1+ cells declined with increasing duration of MS disease progression (Figure 5(g) and (h); *p* < 0.0001). Collinearity of duration of progression with age was not observed and there was no difference in Stro-1 expression between samples isolated from patients with primary or secondary progressive disease.

### MS MSCs display accelerated senescence in vitro

At passages p7, p10 and p12 β-galactosidase staining was performed to visualise senescent cells (control: *n* = 8; MS: *n* = 15). As expected, the percentage of blue, senescent cells increased with *in* vitro passage number for MSCs from both control and individuals with MS (Wilcoxon matched-pairs *t*-test, *p* < 0.0001). Independent effects of age (*p* = 0.024), presence of MS (*p* = 0.02) and passage (*p* < 0.0001) were observed. The effect attributable to disease duration did not reach statistical significance (*p* = 0.063), and there was no difference in β-galactosidase expression between samples isolated from patients with primary or secondary progressive disease.

### MS MSCs have accelerated telomere shortening with expansion in vitro

DNA was isolated from control MSCs (*n* = 6) and MS MSCs (*n* = 10) at passage 2 and 6 to facilitate measurement of telomere length. The number of days in culture was constant between samples isolated for MS (p2 mean = 30 days; p6 mean = 105.3 days) and control MSCs (p2 mean = 30.6 days; p6 mean = 106.2 days) (Mann Whitney’s test: p2, *p* = 0.79; p6, *p* = 0.87).

**Figure 6.**
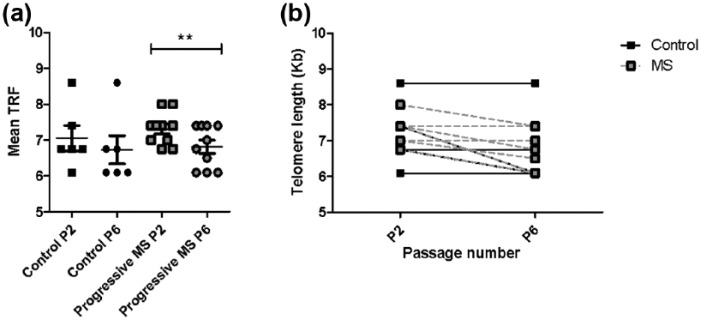
Accelerated telomere shortening in MS MSCs. (a, b) MSCs isolated from patients with progressive MS demonstrate accelerated telomere shortening in vitro. Several samples had identical telomere lengths (**p < 0.01).

As expected, there was a negative association between TRF length and age at both p2 (Pearson’s *r* = −0.537; *p* = 0.032) and p6 (Pearson’s *r* = −0.722; *p* = 0.002). There was no significant difference in TRF between samples isolated from control subjects and those with MS (Figure 6(a)). TRF length also decreased with increasing passage number for both control and MS MSCs, but comparison of TRF between p2 and p6 reached statistical significance only in MS MSCs (paired Student’s *t*-test, *p* = 0.004) consistent with accelerated telomeric loss with ex vivo expansion of MSCs from MS patients (Figure 6(b)). Using multiple regression, significant effects of age (*p* = 0.024) and passage (*p* < 0.0001) were observed but there was no significant association between TRF length and presence of MS, duration of disease progression or subtype of progressive disease.

## Discussion

Treatment of progressive MS represents a major unmet clinical need, and there has been early and rapid translation of bone-marrow-derived cell therapy from in vitro experiments and those employing in vivo models of demyelination to clinical studies.^1,19^ However, few studies have examined the phenotype of MS-patient marrow and MS MSCs in detail.

Carrai et al.^2^ compared bone marrow from patients with non-Hodgkin’s lymphoma and MS and reported reduced cellularity in MS with a trend towards increased fibrosis and reduced matrix metallopeptidase-9 (MMP-9) expression. Others have reported similarity in B- and T-cell populations in marrow aspirates from patients with MS or cardiac disease^9^ although relative increases in IgA^20^ and reduced populations of NK cells^9^ have been noted. Significantly, these studies did not always control for effects of age and/or comparator groups were not always healthy.

Successful isolation of MSCs from patients with MS has previously been reported^3–7^ although, as with studies of marrow, control groups have not always been age-matched and/or disease-free. Broadly, similar patterns of mesenchymal differentiation have been reported,^4–7^ but only De Oliveira et al.^7^ reported increased senescence with alterations in the MS MSC secretome (reduced interleukin-10 (IL-10) and transforming growth factor-beta (TGF-β)) and altered gene transcription. Interestingly, they also demonstrated reduced MS MSC-mediated anti-proliferative effects on co-culture with allogeneic T-lymphocytes. In this study, the subjects were young and well age-matched.

We are aware of only one other study examining telomere length in bone marrow-derived cells in MS, and this was in circulating leukocytes rather than MSCs; shortened TRFs were noted in peripheral blood mononuclear cells isolated from patients with primary progressive MS, particularly those with severe disabilty.^21^

The recent acknowledgement of the reparative potential of MSCs for a wide variety of pathological conditions has renewed interest in the importance of the stromal compartment of bone marrow. However, more recently still, it has been recognised that age,^22,23^ time in vitro^24^ and disease states^8^ can influence stromal cell function, although whether these changes contribute to reduced reparative potential of MSCs and pathogenesis, or occur as phenomena associated with ageing and/or consequence of disease is not always clear.

Here, we show that the bone marrow niche is indeed altered in MS and MSCs isolated from patients with MS have reduced ex vivo expansion potential and show markers of premature ageing *in vitro*. Furthermore, allowing for effects of age, expansion potential of MS MSCs declines in association with duration of progressive MS.

Our results corroborate and extend the findings of those which have previously reported decreased cellularity of marrow,^2^ reduced expansion potential^7^ and shortening of TRF length in bone marrow-derived cells isolated from patients with progressive MS. Furthermore, they highlight the importance of carefully controlling for age and time in vitro in related studies. We show for the first time that MS marrow is phenotypically abnormal with the striking finding of T-cell nodules in particular. Although definitive identification of MSCs in vivo is challenging, reduced numbers of Stro-1-positive cells and expression of p16 in a predominantly nuclear distribution within spindle-shaped, vimentin-positive cells in hypocellular areas support the hypothesis that stromal support for haematopoiesis is impaired in MS-patient marrow.

This study reports limited data on functionality of MSCs isolated from patients with MS including only proliferation and clonogenic potential, as well as mesenchymal differentiation potential. Nonetheless, our findings of reduced expansion potential and premature senescence of MS MSCs in vitro have clear implication for trial protocols employing expansion of MSCs for autologous infusion in MS and other conditions, perhaps most particularly those where oxidative stress is implicated in the pathophysiology.^21^

The findings of changes consistent with accelerated ageing in cells of bone marrow origin in MS are of potential significance to the pathophysiology of progressive MS. Although MS is typically considered a disease of young adults, progressive disease driven by axonal loss is the major determinant of disability^25^ and this is clearly influenced by age; the onset of progressive disease typically occurs around 45 years, irrespective of the MS subtype^26^ and, with advancing age patients are more likely to have progressive disease.^27,28^ The reasons underlying the influence of age on disease course remain unclear although note has been made of age-associated failure of remyelination,^29^ as well as chronic immune system activation, reduction in production of trophic factors and exhaustion of compensatory mechanisms within the central nervous system (CNS; reviewed by Larochelle et al.^30^). Furthermore, processes known to occur in normal ageing including genomic instability, mitochondrial dysfunction, telomere attrition, protein misfolding, deregulated nutrient sensing and cellular senescence^31^ have also been implicated in MS pathogenesis, and the possibility that progressive MS may be driven by neurodegenerative, age-related mechanisms has previously been raised.^28,32^ Although chronic inflammation could contribute to premature ageing, patients with MS who have been exposed to the most potent immunosuppressive treatments are not protected from progressive disease, suggesting that there are additional contributory factors.^33^

A potential limitation of our study is the difference in MSC origin between control subjects (proximal femur) and patients with MS (posterior iliac crest). However, pelvic marrow is generally accepted as the gold standard for isolation of MNCs and MSCs,^34–36^ so reduction in MSC expansion potential seen in patients with progressive MS in our study is likely to underestimate the magnitude of changes between control and MS MSCs. A possible confounding effect of the presence of osteoarthritis in the control cohort is also acknowledged although, beyond age-related effects, a consistent effect of osteoarthritis on isolation and proliferation of MSCs from femoral shaft marrow has not been reported.^37–40^

Although some patients with secondary progressive MS had previously been exposed to immunomodulatory disease-modifying agents, only two samples with a history of exposure to DMTs were included in the analysis of MSC phenotype in vitro. None of the patients with primary progressive disease had received DMTs, and there was no differential effect of disease subtype on any of the parameters examined. This would suggest that the observed, disease-specific effects are unlikely to be attributable to effects of prior exposure to DMTs.

Additional investigation will be required to explore further the mechanisms underlying the observed impairments in MS MSC in vitro expansion potential and determine whether MS MSCs have trophic, neuroglial protective and immunoregulatory functions equivalent to those of MSCs isolated from control subjects. It is anticipated that these functions may be impaired in MS MSCs and will require correction if cell-based therapies for the treatment of MS are to be optimised. While use of allogenic MSCs may be an option, clarification of the mechanisms underlying our findings will contribute to the understanding of the pathophysiology of progressive MS and may facilitate identification of novel therapeutic interventions, not limited to those employing cell-based approaches. These findings are also likely to be of relevance to the treatment of other neurodegenerative and autoimmune diseases, perhaps most particularly those where chronic inflammation and oxidative stress play key pathological roles.

## Supplementary Material

Supplementary material

## References

[1] Rice CM Kemp K Wilkins A et al Cell therapy for multiple sclerosis: An evolving concept with implications for other neurodegenerative diseases. Lancet 2013; 382(9899): 1204–1213. 24095194 10.1016/S0140-6736(13)61810-3

[2] Carrai V Donnini I Mazzanti B et al Immunohistochemistry analysis of bone marrow biopsies in multiple sclerosis patients undergoing autologous haematopoietic stem cells transplantation. Clin Neurol Neurosurg 2013; 115: 1044–1048. 23218837 10.1016/j.clineuro.2012.10.032

[3] Papadaki HA Tsagournisakis M Mastorodemos V et al Normal bone marrow hematopoietic stem cell reserves and normal stromal cell function support the use of autologous stem cell transplantation in patients with multiple sclerosis. Bone Marrow Transplant 2005; 36: 1053–1063. 16205726 10.1038/sj.bmt.1705179

[4] Mazzanti B Aldinucci A Biagioli T et al Differences in mesenchymal stem cell cytokine profiles between MS patients and healthy donors: Implication for assessment of disease activity and treatment. J Neuroimmunol 2008; 199: 142–150. 18562015 10.1016/j.jneuroim.2008.05.006

[5] Mallam E Kemp K Wilkins A et al Characterization of in vitro expanded bone marrow-derived mesenchymal stem cells from patients with multiple sclerosis. Mult Scler 2010; 16: 909–918. 20542920 10.1177/1352458510371959

[6] Harris VK Faroqui R Vyshkina T et al Characterization of autologous mesenchymal stem cell-derived neural progenitors as a feasible source of stem cells for central nervous system applications in multiple sclerosis. Stem Cells Transl Med 2012; 1: 536–547. 23197858 10.5966/sctm.2012-0015 PMC3659719

[7] De Oliveira GL De Lima KW Colombini AM et al Bone marrow mesenchymal stromal cells isolated from multiple sclerosis patients have distinct gene expression profile and decreased suppressive function compared with healthy counterparts. Cell Transplant 2015; 24: 151–165. 24256874 10.3727/096368913X675142

[8] Wang J Liao L Wang S et al Cell therapy with autologous mesenchymal stem cells-how the disease process impacts clinical considerations. Cytotherapy 2013; 15: 893–904. 23751203 10.1016/j.jcyt.2013.01.218

[9] Jons D Kneider M Fogelstrand L et al Early hematopoiesis in multiple sclerosis patients. J Neuroimmunol 2016; 299: 158–163. 27725115 10.1016/j.jneuroim.2016.09.004

[10] Rice CM Marks DI Walsh P et al Repeat infusion of autologous bone marrow cells in multiple sclerosis: Protocol for a phase I extension study (SIAMMS-II). BMJ Open 2015; 5: e009090. 10.1136/bmjopen-2015-009090 PMC4567673 26363342

[11] Rice CM Marks DI Ben-Shlomo Y et al Assessment of bone marrow-derived Cellular Therapy in progressive Multiple Sclerosis (ACTiMuS): Study protocol for a randomised controlled trial. Trials 2015; 16: 463. 26467901 10.1186/s13063-015-0953-1 PMC4606493

[12] Guttridge MG Belfield H Hollyman D et al An internal positive control for the enumeration of CD45(+) and CD34(+) cells by flow cytometry allows monitoring of reagent and operator performance. Cytotherapy 2007; 9: 275–282. 17464759 10.1080/14653240701247846

[13] Kemp K Hares K Mallam E et al Mesenchymal stem cell-secreted superoxide dismutase promotes cerebellar neuronal survival. J Neurochem 2010; 114: 1569–1580. 20028455 10.1111/j.1471-4159.2009.06553.x

[14] Dominici M Le Blanc K Mueller I et al Minimal criteria for defining multipotent mesenchymal stromal cells. The International Society for Cellular Therapy position statement. Cytotherapy 2006; 8: 315–317. 16923606 10.1080/14653240600855905

[15] Orazi A O’Malley D Arber D. Illustrated pathology of the bone marrow. Cambridge: Cambridge University Press, 2006.

[16] Kresnik PK Krasna M Rozman P et al Collection and immunoselection of CD34+ cells: The impact of age, sex, and diabetes in patients with chronic heart failure. Transfusion 2016; 56: 1792–1800. 27185200 10.1111/trf.13646

[17] Wagner W Horn P Castoldi M et al Replicative senescence of mesenchymal stem cells: A continuous and organized process. PLoS ONE 2008; 3: e2213. 18493317 10.1371/journal.pone.0002213 PMC2374903

[18] Simmons PJ Torok-Storb B. Identification of stromal cell precursors in human bone marrow by a novel monoclonal antibody, STRO-1. Blood 1991; 78: 55–62. 2070060

[19] Uccelli A Laroni A Freedman MS. Mesenchymal stem cells as treatment for MS – Progress to date. Mult Scler 2013; 19: 515–519. 23124791 10.1177/1352458512464686

[20] Fredrikson S Baig S Link H. Immunoglobulin producing cells in bone marrow and blood of patients with multiple sclerosis and controls. J Neurol Neurosurg Psychiatry 1991; 54: 412–414. 1713951 10.1136/jnnp.54.5.412 PMC488539

[21] Guan JZ Guan WP Maeda T et al Patients with multiple sclerosis show increased oxidative stress markers and somatic telomere length shortening. Mol Cell Biochem 2015; 400: 183–187. 25424527 10.1007/s11010-014-2274-1

[22] Stolzing A Jones E McGonagle D et al Age-related changes in human bone marrow-derived mesenchymal stem cells: Consequences for cell therapies. Mech Ageing Dev 2008; 129: 163–173. 18241911 10.1016/j.mad.2007.12.002

[23] Beane OS Fonseca VC Cooper LL et al Impact of aging on the regenerative properties of bone marrow-, muscle-, and adipose-derived mesenchymal stem/stromal cells. PLoS ONE 2014; 9: e115963. 25541697 10.1371/journal.pone.0115963 PMC4277426

[24] Von Bahr L Sundberg B Lonnies L et al Long-term complications, immunologic effects, and role of passage for outcome in mesenchymal stromal cell therapy. Biol Blood Marrow Transplant 2012; 18: 557–564. 21820393 10.1016/j.bbmt.2011.07.023

[25] Scalfari A Neuhaus A Degenhardt A et al The natural history of multiple sclerosis, a geographically based study 10: Relapses and long-term disability. Brain 2010; 133: 1914–1929. 20534650 10.1093/brain/awq118 PMC2892939

[26] Tutuncu M Tang J Zeid NA et al Onset of progressive phase is an age-dependent clinical milestone in multiple sclerosis. Mult Scler 2013; 19: 188–198. 22736750 10.1177/1352458512451510 PMC4029334

[27] Minden SL Frankel D Hadden LS et al Disability in elderly people with multiple sclerosis: An analysis of baseline data from the Sonya Slifka Longitudinal Multiple Sclerosis Study. NeuroRehabilitation 2004; 19: 55–67. 14988588

[28] Scalfari A Neuhaus A Daumer M et al Age and disability accumulation in multiple sclerosis. Neurology 2011; 77: 1246–1252. 21917763 10.1212/WNL.0b013e318230a17d PMC3179646

[29] Sim FJ Zhao C Penderis J et al The age-related decrease in CNS remyelination efficiency is attributable to an impairment of both oligodendrocyte progenitor recruitment and differentiation. J Neurosci 2002; 22: 2451–2459. 11923409 10.1523/JNEUROSCI.22-07-02451.2002 PMC6758320

[30] Larochelle C Uphaus T Prat A et al Secondary progression in multiple sclerosis: Neuronal exhaustion or distinct pathology? Trends Neurosci 2016; 39: 325–339. 26987259 10.1016/j.tins.2016.02.001

[31] Lopez-Otin C Blasco MA Partridge L et al The hallmarks of aging. Cell 2013; 153: 1194–1217. 23746838 10.1016/j.cell.2013.05.039 PMC3836174

[32] Confavreux C Vukusic S. Age at disability milestones in multiple sclerosis. Brain 2006; 129: 595–605. 16415309 10.1093/brain/awh714

[33] Coles AJ Cox A Le Page E et al The window of therapeutic opportunity in multiple sclerosis: Evidence from monoclonal antibody therapy. J Neurol 2006; 253: 98–108. 16044212 10.1007/s00415-005-0934-5

[34] Davies BM Snelling SJ Quek L et al Identifying the optimum source of mesenchymal stem cells for use in knee surgery. J Orthop Res. Epub ahead of print 9 12 2016 DOI: 10.1002/jor.23501. 27935105

[35] Narbona-Carceles J Vaquero J Suarez-Sancho S et al Bone marrow mesenchymal stem cell aspirates from alternative sources: Is the knee as good as the iliac crest? Injury 2014; 45(suppl. 4): S42–S47. 25384474 10.1016/S0020-1383(14)70009-9

[36] Hyer CF Berlet GC Bussewitz BW et al Quantitative assessment of the yield of osteoblastic connective tissue progenitors in bone marrow aspirate from the iliac crest, tibia, and calcaneus. J Bone Joint Surg Am 2013; 95: 1312–1316. 23864180 10.2106/JBJS.L.01529

[37] Garcia-Alvarez F Alegre-Aguaron E Desportes P et al Chondrogenic differentiation in femoral bone marrow-derived mesenchymal cells (MSC) from elderly patients suffering osteoarthritis or femoral fracture. Arch Gerontol Geriatr 2011; 52: 239–242. 20416958 10.1016/j.archger.2010.03.026

[38] Jones E English A Churchman SM et al Large-scale extraction and characterization of CD271+ multipotential stromal cells from trabecular bone in health and osteoarthritis: Implications for bone regeneration strategies based on uncultured or minimally cultured multipotential stromal cells. Arthritis Rheum 2010; 62: 1944–1954. 20222109 10.1002/art.27451

[39] Scharstuhl A Schewe B Benz K et al Chondrogenic potential of human adult mesenchymal stem cells is independent of age or osteoarthritis etiology. Stem Cells 2007; 25: 3244–3251. 17872501 10.1634/stemcells.2007-0300

[40] Murphy JM Dixon K Beck S et al Reduced chondrogenic and adipogenic activity of mesenchymal stem cells from patients with advanced osteoarthritis. Arthritis Rheum 2002; 46: 704–713. 11920406 10.1002/art.10118

