# Supplementary material for: Reduced cellularity of bone marrow in multiple sclerosis with decreased MSC expansion potential and premature ageing in vitro
Source: Mult Scler. 2017 May 26;24(7):919–31. doi: 10.1177/1352458517711276 (PMC6029147; doi:10.1177/1352458517711276)
Supplement: Supplementary material [file MSJ711276_supplementary_tables.pdf]

SUPPLEMENTARY INFORMATION

Table 1 Summary data for both control and MS cohorts including information regarding which samples were included for each of the analyses undertaken

Subject	Sex	Age	Classification MS	Duration of progression of MS (years)	Exposure to disease modifying therapy	Experiments including analysis of samples
Control 1	M	54	N/A	N/A	N/A	Phenotype, PDT,CFU,B-GAL
Control 2	F	65	N/A	N/A	N/A	Phenotype, PDT,CFU,B-GAL
Control 3	M	70	N/A	N/A	N/A	Phenotype, PDT,CFU,B-GAL
Control 4	F	60	N/A	N/A	N/A	Phenotype, PDT,CFU,B-GAL + Telomere length
Control 5	M	49	N/A	N/A	N/A	Phenotype, PDT,CFU,B-GAL + Telomere length
Control 6	M	66	N/A	N/A	N/A	Phenotype, PDT,CFU,B-GAL
Control 7	F	68	N/A	N/A	N/A	Phenotype, PDT,CFU,B-GAL
Control 8	M	58	N/A	N/A	N/A	Phenotype, PDT,CFU,B-GAL + Telomere length
Control 9	M	55	N/A	N/A	N/A	Telomere length
Control 10	M	59	N/A	N/A	N/A	Telomere length
Control 11	F	53	N/A	N/A	N/A	Telomere length
**Mean (yrs)**		**59.73**				
**Median (yrs)**		**59**				
MS 1	M	48	PP	4	None	Phenotype, PDT,CFU,B-GAL + Telomere length + Trephine
MS 2	F	48	PP	15	None	Phenotype, PDT,CFU,B-GAL
MS 3	M	60	SP	10	None	Phenotype, PDT,CFU,B-GAL + Telomere length
MS 4	M	33	SP	3	Beta-interferon	Phenotype, PDT,CFU,B-GAL + Trephine
MS 5	F	47	PP	9	None	Phenotype, PDT,CFU,B-GAL + Telomere length + Trephine
MS 6	F	47	PP	6	None	Phenotype, PDT,CFU,B-GAL +Trephine
MS 7	F	59	SP	15	None	Phenotype, PDT,CFU,B-GAL + Telomere length + Trephine
MS 8	M	55	SP	2	None	Phenotype, PDT,CFU,B-GAL + Telomere length + Trephine
MS 9	M	56	SP	15	None	Phenotype, PDT,CFU,B-GAL + Telomere length + Trephine
MS 10	F	53	SP	3	None	Phenotype, PDT,CFU,B-GAL + Telomere length + Trephine
MS 11	M	49	PP	14	None	Phenotype, PDT,CFU,B-GAL + Telomere length + Trephine
MS 12	M	64	PP	15	None	Phenotype, PDT,CFU,B-GAL + Telomere length + Trephine
MS 13	M	50	PP	2	None	Telomere length + Trephine
MS 14	F	52	PP	15	None	Trephine
MS 15	F	40	SP	8	None	Trephine
MS 16	M	50	SP	2	None	Trephine
MS 17	M	63	SP	12	Beta-interferon	Trephine

MS 18	M	55	SP	5	None	Trephine
MS 19	F	41	SP	2	Glatiramer	Trephine
MS 20	F	50	PP	4	None	Trephine
MS 21	F	49	SP	4	Beta-interferon	Trephine
MS 22	F	58	PP	10	None	Trephine
MS 23	F	57	SP	3	Glatiramer	Trephine
MS 24	F	62	SP	8	Beta-interferon	Trephine
MS 25	F	54	SP	2	Copaxone, beta-interferon, alemtuzumab	Trephine
MS 26	M	48	SP	9	None	Phenotype, PDT,CFU,B-GAL
MS 27	M	57	SP	11	None	Phenotype, PDT,CFU,B-GAL
MS 28	F	47	SP	14	Glatiramer, beta-interferon	Phenotype, PDT,CFU,B-GAL
**Mean (yrs)**		**51.86**		**7.93**		
**Median (yrs)**		**51**		**8**		

Table 2 Source and dilution of antibodies for immunohistochemistry of bone marrow trephines and MSC immunocytochemistry

Antibody	Dilution	Manufacturer
CD3	1:100	Leica
CD20	1:300	Leica
CD138	1:100	Dako
Vimentin	1:800	Dako
CD34	1:1500	Leica
P53	1:300	Leica
P16	Pre-dilute	Roche (CINtec)
CD56 (NCAM)	1:200	Leica
CD117	1:500	Dako
CD61	1:200	Leica
Myeloperoxidase	Pre-dilute	Leica
Glycophorin A (CD235a)	1:400	Dako
Ki67	Pre-dilute	Leica
CD73	1:200	Abcam
CD105	1:200	Abcam
CD271	1:200	Millipore
STRO1	1:200	Millipore
Fibronectin	1:400	Sigma
βIII tubulin	1:400	Sigma

Reporting criteria for bone marrow trephines

Samples were reported according to standard clinical methodology including an assessment of myeloid:erythroid ratio (M:E), fibrosis and bone structure. To allow for age-related changes in cellularity,[1, 2] the following were considered normal: <40 years old, 60% cellularity; 40-50 years old, 50% cellularity; 50-60 years old, 40% cellularity; 60-70 years old, 30% cellularity. Immunohistochemistry staining was evaluated on 2 occasions by the same pathologist blinded as to their original assessment.

Proliferation was assessed using immunohistochemistry for the nuclear marker Ki67 across all cell types (myeloid precursors, erythroid cells and megakaryocytes) and <60% total cells positive for Ki67 was considered abnormal.[3]

Expression of p16 (cyclin-dependent kinase inhibitor 2A, multiple tumor suppressor 1) staining was analysed semiquantitatively; + representing 1-4 positive cells, ++ 5-10 positive cells and +++ 11-15 positive cells per high power field (x40 magnification).

1. Bain BJ. The bone marrow aspirate of healthy subjects. *Br J Haematol*. 1996; 94: 206-9.
2. Orazi A, O'Malley D and Arber D. Illustrated Pathology of the Bone Marrow. Cambridge University Press, 2006.
3. Pellegrini W, Facchetti F, Marocolo D, et al. Assessment of cell proliferation in normal and pathological bone marrow biopsies: a study using double sequential immunophenotyping on paraffin sections. *Histopathology*. 1995; 27: 397-405.
